# Temporal patterns of physical activity and sedentary behavior in 10–14 year-old children on weekdays

**DOI:** 10.1186/s12889-015-2093-7

**Published:** 2015-08-19

**Authors:** Stijn De Baere, Johan Lefevre, Kristine De Martelaer, Renaat Philippaerts, Jan Seghers

**Affiliations:** Department of Kinesiology, KU Leuven, Tervuursevest 101, 3001 Leuven, Belgium; Faculty of Physical Education and Physiotherapy, Vrije Universiteit Brussel, Pleinlaan 2, 1050 Brussels, Belgium; Department of Movement and Sports Sciences, Ghent University, Watersportlaan 2, 9000 Ghent, Belgium

**Keywords:** SenseWear Mini Armband, Electronic activity diary, Youth, Time-use, Segmentation

## Abstract

**Background:**

An important but often ignored aspect of physical activity (PA) and sedentary behavior (SB) is the chronological succession of activities, or temporal pattern. The main purposes of this study were (1) to investigate when certain types of PA and SB compete against each other during the course of the day and (2) compare intensity- and domain-specific activity levels during different day-segments.

**Methods:**

The study sample consists of 211 children aged 10–14, recruited from 15 primary and 15 secondary schools. PA was assessed combining the SenseWear Mini Armband (SWM) with an electronic activity diary. The intensity- and domain-specific temporal patterns were plotted and PA differences between different day-segments (i.e., morning, school, early evening and late evening) were examined using repeated-measures ANCOVA models.

**Results:**

Physical activity level (PAL) was highest during the early evening (2.51 MET_SWM_) and school hours (2.49 MET_SWM_); the late evening segment was significantly less active (2.21 MET_SWM_) and showed the highest proportion of sedentary time (54 % of total time-use). Throughout the different day-segments, several domains of PA and SB competed with each other. During the critical early-evening segment, screentime (12 % of time-use) and homework (10 %) were dominant compared to activity domains of sports (4 %) and active leisure (3 %). The domain of active travel competed directly with motor travel during the morning (5 % and 6 % respectively) and early-evening segment (both 8 %).

**Conclusions:**

Throughout the day, different aspects of PA and SB go in competition with each other, especially during the time period immediately after school. Detailed information on the temporal patterns of PA and SB of children could help health professionals to develop more effective PA interventions and promotion strategies. By making adaptations to the typical day schedule of children (e.g., through the introduction of extra-curricular PA after school hours), their daily activity levels might improve.

**Electronic supplementary material:**

The online version of this article (doi:10.1186/s12889-015-2093-7) contains supplementary material, which is available to authorized users.

## Background

The beneficial health effects of regular physical activity (PA) on children’s body composition, cardiovascular risk factors, bone health and mental well-being are well known [1–4]. Nevertheless, longitudinal evidence has shown that activity levels decline throughout childhood and adolescence [5–8]. Certain key periods in life coincide with important changes in PA behavior. In children, the period of the school-stage transition from primary to secondary school has shown to exert a considerable negative impact on the activity behavior of children [9–11]. Dumith et al. [5] estimated the average decline of PA levels at 7 % per year, starting in early adolescence. While PA levels decrease, levels of sedentary behavior (SB) appear to increase as children get older [12]. A longitudinal study on a sample of Norwegian children showed that the proportion of all screen-based sedentary behaviors (i.e., the use of television/DVD and use of computer/videogames) had increased significantly after the school-stage transition [13]. For girls, the decreasing activity level might be even more worrying, as epidemiological evidence has consistently pointed out that girls are less active compared with boys across all ages [14, 15]. A study on a sample of children from various European countries indicated that the overall PA level of boys in terms of activity counts per day was 21 % higher at the age of 9 and 26 % higher at the age of 15 compared with girls [16].

There is clearly a need for effective PA interventions that address the decreasing activity levels of girls and boys, especially around the ages of the school-stage transition. Accurate epidemiological information providing a comprehensive image of the current patterns of PA and SB is required as a solid basis to develop interventions and evaluate their efficacy [17]. Consequently, PA patterns of children should ideally be assessed and examined in its totality, based on the FITT-principle: including the frequency, intensity, time and type dimension of PA [18, 19]. In general, the time dimension of the FITT-principle is interpreted as the duration of the activities. However, besides duration it can also refer to the chronological succession of the activities, also known as the temporal pattern. By investigating the temporal pattern of activities we can better understand the typical day schedule of children and identify the timeframes when PA and SB compete against each other [20]. Despite the potential impact of the temporal pattern on the activity levels of children, studies that have investigated this aspect of PA and SB are limited.

Biddle et al. [20] have published an interesting study on the temporal patterns of leisure-time PA and SB in 13-16 year-old UK adolescents. In their study, the authors described and examined when certain behaviors occurred throughout the day along with the setting of PA and SB, using a combination of two self-report measures. These days, however, accelerometry has become the unofficial norm when assessing PA levels under free-living conditions in children. The use of accelerometers has substantially improved our ability to accurately quantify the frequency, intensity and duration of children’s PA and SB [21]. However, if we aim to capture all the aspects of the temporal pattern, accelerometry on its own is insufficient, since it does not provide information on the behavioral domain wherein activities of children occur (e.g., school, sports, transport, screen-based activities, etc.).

In the current study, the temporal pattern of PA and SB behaviors in 10–14 year-old children (i.e., last 2 years of primary and first 2 years of secondary school) is investigated by combining the SenseWear Mini Armband (SWM) with an electronic activity diary. Consequently, we could simultaneously register all 4 dimensions of PA and SB, as well as the temporal succession of the activities. This study will focus exclusively on weekdays during school periods, because the chronological succession of activities on weekend days shows almost no organized time-structure in children. The main purposes are (1) to investigate when certain types of PA and SB compete against each other during the course of the day and (2) compare different day-segments with respect to intensity- and domain-specific PA levels. As a secondary purpose the temporal patterns and activity levels of PA and SB on weekdays were compared between sexes and school stages.

## Methods

### Participants and recruitment

A total of 241 children (122 boys and 119 girls) aged between 10 and 14 years were recruited from 15 primary and 15 secondary schools in Flanders, Belgium. Schools were randomly selected based on a realistic stratification across the 3 different Flemish school networks in both rural and urban areas. Sixty-one percent of all schools that were approached approved to participate in the study. Within the schools that consented to take part in the study, an equal amount of boys and girls were randomly recruited in the last two years of primary or the first two years of secondary school, with an average of 8 children per school. Both children and their parents were informed about the study aims, study protocol, benefits and risks. The Medical Ethics Committee of the KU Leuven approved the study and written informed consent was obtained from both children and parents before the start of the data collection. Data were collected evenly throughout the year except during school holidays. The recruited sample from the current study is the same as in a previous study by De Baere et al. [22].

### Anthropometry

Anthropometrics were assessed shoeless and in light clothing. Stature and sitting height were measured to the nearest 0.1 cm using a portable anthropometer, body weight was determined to the nearest 0.1 kg using a digital scale. Weight status was determined using sex and age specific BMI standards of Flemish children [23]. An estimate of biological maturity was obtained using the regression equations of Mirwald et al. [24]. These equations estimate the time to peak height velocity (PHV) using chronological age, sex, sitting height, stature and body weight. A negative PHV value represents the number of years a child is from PHV, a positive value the number of years a child is beyond PHV.

### Assessment of physical activity

The SWM (Bodymedia, Inc, Pittsburg, PA, USA) is a multi-sensor activity monitor that combines tri-axial accelerometry with physiological measures (i.e., skin temperature, heat flux and galvanic skin response) for the estimation of several activity parameters. The information from the different sensors together with personal characteristics of the participant (i.e., sex, age, stature, body weight and handedness) is processed through proprietary algorithms from the SenseWear software (SenseWear Professional software version 7.0) to estimate energy expenditure, PA intensity and number of steps on a minute-by-minute basis. A validation study of the SWM by Calabro et al. [25] in a sample of 10–16 year-old children showed that the SWM provides reasonably accurate estimates of energy expenditure under free-living conditions. Participants were asked to wear the SWM on the left upper arm for 7 consecutive days, 24 h per day and were instructed to only remove the armband during water-based activities. A 7-day accelerometer PA monitoring protocol is known to provide reliable estimates of habitual PA behavior in children [26].

In addition to the continuous assessment through the SWM, participants were instructed to record their activities in an electronic activity diary that was developed at the Department of Kinesiology of the KU Leuven. Through the electronic activity diary, information on the activity type or behavioral domain of the activities is provided. The activity diary software program is integrated in a Palm Z22 handheld computer (Palm, Inc., Sunnyvale, CA, USA) and was initially developed for a study on adults [27]. The adult diary was adapted with regard to diary content and language to meet the needs of our target group and the feasibility of the electronic diary was tested in 10–14 year-old children. The diary for children comprises 7 main categories: school, eating and drinking, personal care, household chores, sleep, transportation and leisure time. The main category of transportation is subdivided into walking, cycling and motorized travel. The main category of leisure time is subdivided into a series of active and inactive pastimes (e.g., television viewing, reading, active play, sport participation, etc.). At the beginning of every new activity, participants had to register their actions into the diary program, except during school hours. Information on the school schedule was obtained from the different school administrations. The real-time assessment strategy of the activity diary program has proven to provide more accurate behavioral information in comparison with the conventional retrospective assessment strategies, since detailed behavioral information is entered into the device at the time of occurrence [28]. These real-time assessment strategies are particularly advantageous in a young population, given the intermittent nature of their activity behavior and cognitive immaturity [29, 30]. The data of the SWM and electronic activity diary were downloaded and the activity outputs of both devices were time-merged based on the internal clocks of both devices. Consequently, 24 h information on all 4 dimensions of PA (i.e., frequency, intensity, time and type) was available on a minute-by-minute basis. In some cases, missing values from the SWM were imputed using the activity-type information from the electronic diary. Missing data for sleep were replaced with the average energy cost for sleep during all other nights. Missing data for personal care and swimming were substituted with the MET-value and corresponding energy expenditure from the compendium of PA for youth by Ridley et al. [31].

Based on the combined SWM and electronic diary information, a series of activity variables was created. Physical activity level (PAL) is a measure of daily energy expenditure and was computed as the average MET-value provided by the SWM (MET_SWM_) during 24 h. Time variables for the different intensity levels were calculated based on SWM intensity thresholds for children: sedentary (≤1.8 MET_SWM_), light (>1.8 – ≤ 5.1 MET_SWM_), moderate (>5.1 – ≤ 7.2 MET_SWM_) and vigorous (>7.2 MET_SWM_) activities [22]. These SWM intensity thresholds were established using a structured indirect calorimetry protocol. For example, the threshold between sedentary behavior and light PA was identified based on two criteria: correct identification of sedentary activities (i.e., sensitivity) and correct exclusion of light intensity activities (i.e., specificity). The threshold that showed the lowest classification error (1.8 MET_SWM_) was then considered the optimal threshold. Periods of sleep were distinguished from the 4 intensity categories using the data from the electronic activity diary. Activity-type information from the electronic diary combined with activity-intensity information from the SWM was used to classify minute-by-minute PA data into 10 behavioral domains. The behavioral domain of school activities consists of all activities that were executed during school time, including theoretic classes, recess time, lunch breaks and physical education classes. Sport participation entails all the organized and non-organized sport activities children engage in during leisure time with an intensity level above sedentary. The active leisure domain consists of active behaviors during leisure time (e.g., playing outdoors, active hobbies, shopping) with an intensity level above sedentary, excluding sport activities. Transfers on foot or by bike with an intensity level above sedentary are part of the active travel domain. Homework comprises all the sedentary school-related activities performed after school hours. Screentime consists of computer or tablet use, watching television and video games at sedentary intensity, but does not include screentime for homework. The inactive leisure domain encompasses all the sedentary activities during leisure time, screentime excluded (e.g., reading, inactive hobbies, social interactions with peers). The domain of motor travel comprises all transfers children make by car, bus, train or any other motorized vehicle. Eating and drinking, personal care and household chores are part of the domain of common activities of daily life (CADL). Finally, the category ‘other’ are all the minutes that could not be allocated to one of the 10 behavioral domains due to missing data or contradictory activity-type and intensity information.

For the analysis of the temporal pattern, regular weekdays (i.e., Monday, Tuesday, Thursday and Friday) were divided into different day-segments: morning (6:00–9:00), school (9:00–15:00), early evening (15:00–18:00), late evening (18:00–21:00) and night (21:00–6:00). Since children in Flanders only go to school during the forenoon on Wednesdays, the time range of 9:00 to 15:00 was divided into the day-segments of school (9:00–12:00) and afternoon (12:00–15:00).

Participants were included in the analysis if data of at least 4 valid weekdays were available: including at least 3 regular weekdays (i.e., Monday, Tuesday, Thursday, Friday) and Wednesday. According to Scheers et al. [32], 3 days of SenseWear monitoring from Monday to Friday are required in an adult population to achieve reliable estimates of PA patterns during weekdays. In the current study, Wednesday’s had to be included for each participant, because Wednesday’s schedule for children differs considerably from other weekdays in Flanders. A valid monitoring day was defined as a day with at least 90 % compliance or 1296 min of combined SWM and activity diary output, after imputation of known activities. This 90 % threshold is based on a comparison with the more often used 600 min per day criteria for accelerometers that are worn only during waking hours [7, 33]. The current 90 % criterion is at the least as strict as the 600 min criterions for accelerometers worn during waking hours only.

### Data analysis

Intensity- and domain-specific activity levels were calculated as means and standard deviations. Design effects were calculated for PAL and the 4 intensity categories to determine the effect of nesting on the school-level. Multilevel modeling is recommended when design-effects of 2.00 or more are found, meaning that the school-level has a considerable effect on the activity level of our sample [34, 35]. The design effects on the current data ranged from 0.06 for PAL to 1.92 for light intensity activity. Consequently, ANCOVA models were used to test mean differences in activity levels on weekdays between both sexes and school stages with weight status as covariate. In addition, effect size correlation coefficients (ES-*r*) for the sex and school-stage effects were calculated using the square root of partial eta squared values [36, 37]. The effect size coefficients can be interpreted using the following scale of magnitude: trivial (*r* < 0.1), small (0.1 ≤ *r* < 0.3) moderate (0.3 ≤ *r* < 0.5), large (0.5 ≤ *r* < 0.7), very large (0.7 ≤ *r* > < 0.9) and nearly perfect (*r* ≤ 0.9 < 1) [38]. Figures representing the temporal pattern on regular weekdays and Wednesdays were made for the time range of 6:00 to midnight. These graphs were created for both the PA intensities and behavioral domains. PA differences between day-segments on regular weekdays and Wednesdays were determined using repeated-measures ANCOVA models with weight status as covariate. For a meaningful comparison of the activity parameters between the different day-segments, equal time-segments of 3 h were required. Therefore, the 6 h school-segment on regular weekdays was averaged to a 3 h day-segment before analysis. Tukey’s HSD tests were carried out for post-hoc comparison between day-segments. Statistical significance was set at *P* < 0.05. All statistical analyses were performed using SAS statistical software, version 9.2 (SAS Institute, Cary, NC, USA).

## Results

Personal characteristics and anthropometrics for the total sample, both sexes and both school stages are shown in Table 1. In total, 211 of the initial 241 participants met the compliance criteria. As a consequence, 30 participants (19 boys and 11 girls) were excluded from further analysis. The group of participants that did not meet the compliance criteria did not differ significantly from the actual sample with respect to age and weight-status. Intensity- and domain-specific activity levels on weekdays are presented in Table 2. Differences between sexes and school stages are reported together with the effect-size correlation coefficients. The levels of PA and SB are presented as pooled means and standard deviations, because only time spent at moderate intensity showed a significant sex by school-stage interaction effect (*P* = 0.01, ES-*r* = 0.17). Average daily moderate intensity activity was similar for boys from primary and secondary school (50.8 min/day and 50.7 min/day respectively), however girls from primary school showed a lower level of moderate PA compared with girls from secondary school (28.9 min/day and 42.0 min/day respectively).

**Table 1:** Descriptives and anthropometric characteristics for the total sample, by sex and school stage

	Total	Boys	Girls	Primary	Secondary
No. of children	241	122	119	127	114
No. with valid data	211	103	108	110	101
Age (yr)	12.0 (1.2)	12.1 (1.2)	12.0 (1.2)	11.1 (0.6)	13.1 (0.7)
Stature (cm)	153.8 (10.1)	153.8 (10.1)	153.8 (10.2)	147.5 (7.5)	160.7 (7.9)
Body weight (kg)	43.3 (10.1)	43.0 (9.7)	43.6 (10.5)	38.1 (7.7)	49.1 (9.3)
Age PHV (yr)	−2.08 (0.96)	−2.13 (0.99)	−2.03 (0.94)	−2.81 (0.50)	−1.27 (0.66)
Overweight (%)	11.8	10.7	13.0	13.6	10.0

Anthropometric characteristics are presented as means and standard deviations

*PHV* peak height velocity

**Table 2:** Levels of physical activity and sedentary behavior on weekdays, comparison between sexes and school stages

	Boys	Girls	ES-*r*	Primary	Secondary	ES-*r*
PAL (MET_SWM_)	1.98 (0.21)	1.84 (0.23)^a^	0.32	1.92 (0.22)	1.89 (0.24)	0.10
Steps (n/day)	12230 (3608)	10098 (2616)^a^	0.34	11685 (3438)	10543 (3070)^b^	0.21
Time spent at different intensity levels						
Sedentary (min/day)	399.5 (98.3)	418.0 (100.7)^a^	0.15	357.9 (83.0)	464.6 (85.8)^b^	0.56
Light (min/day)	397.1 (72.0)	390.7 (80.8)	0.07	422.8 (74.0)	362.2 (66.3)^b^	0.41
Moderate (min/day)	50.8 (21.8)	34.7 (19.1)^a^	0.36	38.9 (23.5)	46.6 (19.4)^b,c^	0.15
Vigorous (min/day)	24.4 (17.8)	14.9 (13.8)^a^	0.28	15.1 (12.5)	24.5 (18.9)^b^	0.27
Sleep (min/day)	568.2 (49.3)	581.8 (62.0)	0.09	605.4 (52.9)	542.2 (39.2)^b^	0.56
Time spent at behavioral domains						
School (min/day)	363.2 (38.6)	358.7 (52.8)	0.03	346.5 (46.9)	376.6 (40.4)^b^	0.32
Sport (min/day)	30.5 (27.7)	29.3 (23.2)	0.03	33.3 (25.8)	26.2 (24.6)^b^	0.15
Active leisure (min/day)	23.7 (27.4)	22.0 (27.5)	0.03	24.8 (30.7)	20.6 (13.1)	0.08
Active travel (min/day)	25.2 (22.0)	27.1 (22.9)	0.07	20.7 (14.9)	32.3 (27.3)^b^	0.26
Homework (min/day)	34.2 (24.6)	39.7 (25.7)^a^	0.14	29.6 (18.7)	45.1 (28.9) ^b^	0.32
Screentime (min/day)	131.3 (68.6)	97.2 (56.2)^a^	0.27	98.3 (58.9)	130.9 (66.8)^b^	0.26
Inactive leisure (min/day)	46.1 (32.7)	70.3 (25.1)^a^	0.26	69.6 (47.5)	46.3 (39.4)^b^	0.25
Motor travel (min/day)	37.6 (31.5)	35.1 (31.3)	0.03	32.1 (26.1)	40.6 (35.8)^b^	0.14
CADL (min/day)	108.5 (34.3)	108.0 (34.0)	0.00	107.9 (33.1)	108.7 (35.2)	0.00
Other (min/day)	71.4 (27.0)	70.9 (26.3)	0.00	71.8 (28.6)	70.4 (24.3)	0.03

Results are presented as pooled means and standard deviations (SD)

*CADL* Common activities of daily life, *ES-r* effect size correlation coefficients

^a^significant main effect sex

^b^significant main effect school stage

^c^significant interaction effect. All tests *P* < 0.05

**Fig. 1:**
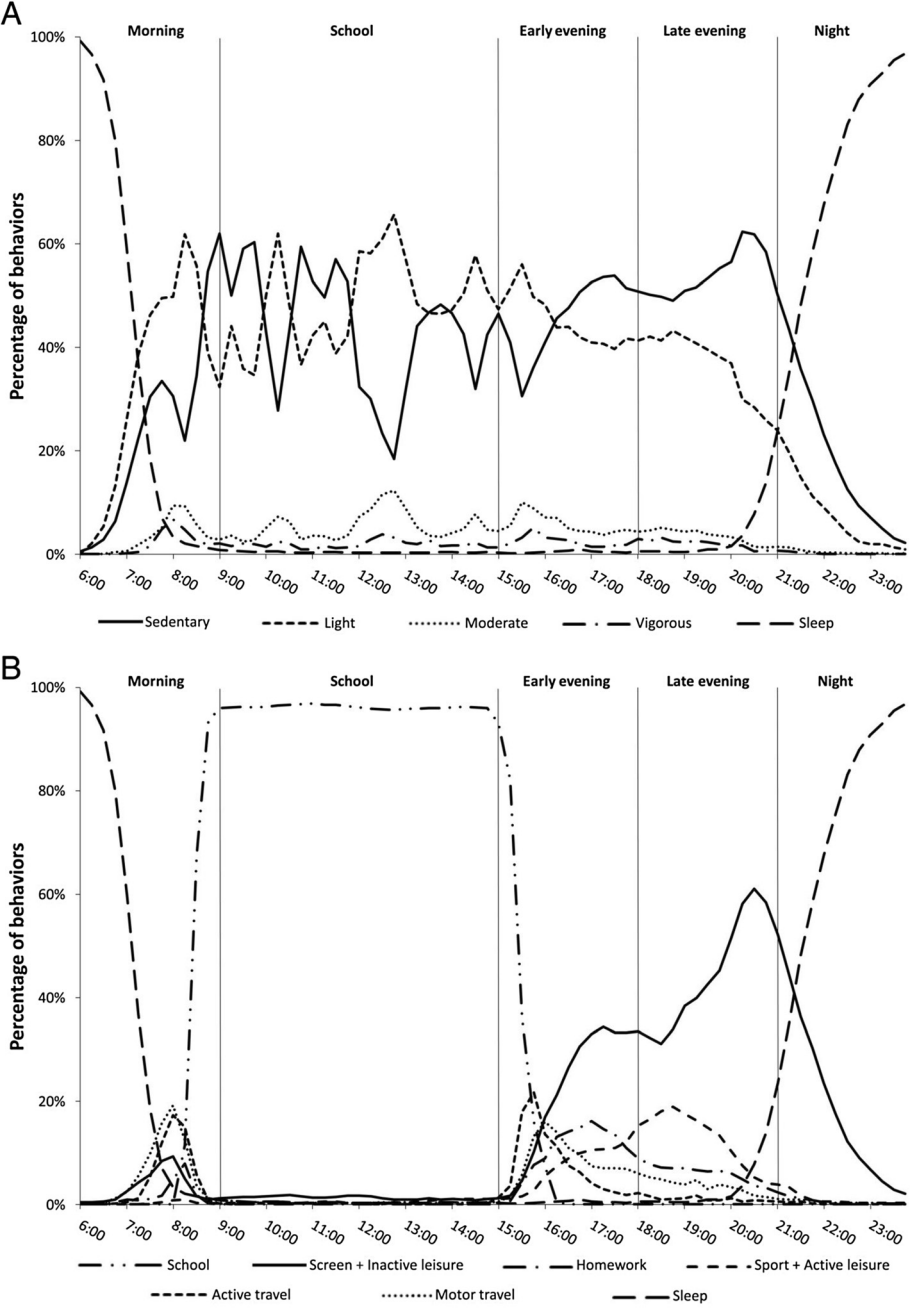
Occurrences of the different intensity levels (Fig. 1a) and behavioral domains (Fig. 1b) as a function of time on regular weekdays

**Fig. 2:**
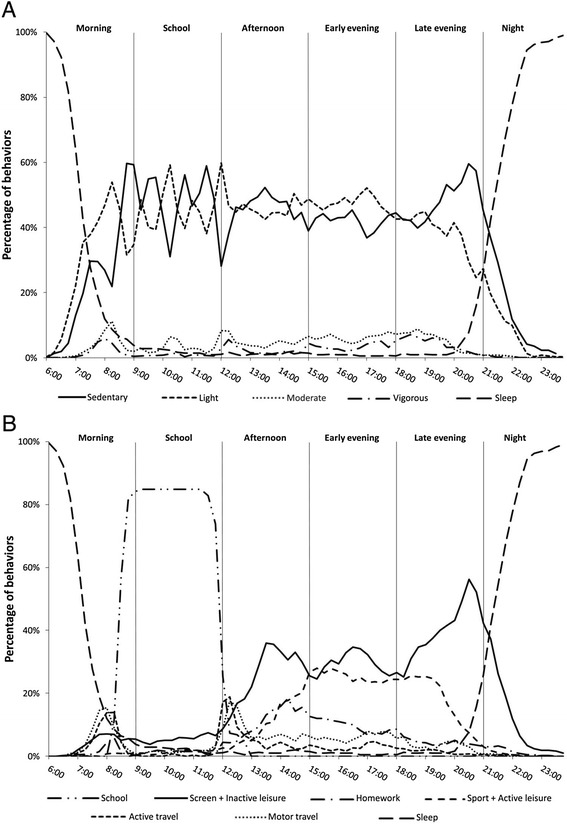
Occurrences of the different intensity levels (Fig. 2a) and behavioral domains (Fig. 2b) as a function of time on Wednesdays

The graphs of the intensity- and domain-specific temporal patterns created per sex and school stage (i.e., boys primary, girls primary, boys secondary and girls secondary), showed little dissimilarity between the 4 subgroups. There were almost no differences with regard to the chronological succession of activity peaks, only the magnitude of the activity peaks showed dissimilarities between sexes and school stages (as illustrated by the activity level differences in Table 2). Therefore, further analysis on the temporal pattern is presented for the total sample only. The temporal graphs for the 4 sex by school stage subgroups can be consulted in the Additional files 1, 2, 3 and 4. Figures 1 and 2 show the temporal pattern for PA intensities and behavioral domains on regular weekdays and Wednesdays. The percentage of time-use for the respective activity intensity and activity domain subcomponents are plotted as a function of the time. PA differences between day-segments for the different PA parameters on regular weekdays (i.e., Monday - Tuesday - Thursday - Friday) are presented in Table 3. The time variables from Table 3 are a quantitative representation of the area under the curve in Fig. 1a (i.e., activity intensities) and 1B (i.e., behavioral domains). Based on the repeated-measures ANCOVA results of PAL and number of steps, school (9:00–15:00) and early evening (15:00–18:00) were the most active day-segments on regular weekdays. With respect to the time spent at different intensity levels, sedentary time was highest during the late evening, no significant difference was found between the school and early evening segment. Light intensity activity was highest during the school hours followed by the early evening, late evening and morning. For moderate intensity, the school and early evening segment showed significantly more minutes spent at moderate intensity compared to the morning and late evening segment. No significant differences were found between the day-segments for vigorous intensity activities. Additional file 5 shows the temporal graph of all separate behavioral domains of PA and SB as a function of time on regular weekdays.

**Table 3:** Comparison of physical activity and sedentary behavior between day-segments on regular weekdays

	Morning		School		Early evening		Late evening		Model	ES-*r*
	(6:00–9:00)		(9:00–15:00)^c^		(15:00–18:00)		(18:00–21:00)		*P*-value	
	Mean (SD)	%	Mean (SD)	%	Mean (SD)	%	Mean (SD)	%		
PAL (MET_SWM_)	2.10 (0.39)		2.49 (0.43)^a^		2.51 (0.5)^a^		2.21 (0.56)		<0.001	0.49
Steps (n)	1519 (618)		2420 (837)^a^		2453 (952)^a^		1893 (1127)		<0.001	0.51
Time spent at different intensity levels										
Sedentary (min)	38 (17)	21	79 (22)^a^	44	83 (25)^a^	46	98 (28)	54	<0.001	0.83
Light (min)	58 (14)	32	87 (19)	48	82 (23)	45	67 (23)	37	<0.001	0.66
Moderate (min)	6 (4)^a^	3	10 (6)^b^	6	11 (7)^b^	6	7 (7)^a^	4	<0.001	0.41
Vigorous (min)	4 (5)^a^	2	4 (4)^a^	2	4 (6)^a^	2	4 (6)^a^	2	0.15	0.09
Sleep (min)	75 (23)	42	0 (0)	0	1 (5)	1	5 (10)	3	<0.001	0.95
Time spent at behavioral domains										
School (min)	26 (9)	14			35 (15)	20	0 (0)	0	<0.001	0.99
Sport (min)	0 (1)	0			7 (12)	4	14 (17)	8	<0.001	0.54
Active leisure (min)	0 (2)	0			6 (11)	3	10 (16)	6	<0.001	0.46
Active travel (min)	9 (9)	5			14 (13)	8	2 (4)	1	<0.001	0.68
Homework (min)	3 (5)	2			18 (15)	10	11 (12)	6	<0.001	0.65
Screentime (min)	3 (7)	2			22 (22)	12	55 (32)	31	<0.001	0.81
Inactive leisure (min)	3 (6)	2			15 (17)	8	24 (25)	13	<0.001	0.60
Motor travel (min)	11 (14)	6			15 (16)	8	7 (8)	4	<0.001	0.55
CADL (min)	39 (13)^a^	21			15 (12)	9	35 (18)^a^	19	<0.001	0.82
Other (min)	12 (9)	7			32 (18)	18	18 (14)	10	<0.001	0.51

Results are presented as means and standard deviations (SD)

*CADL* Common activities of daily life, *ES-r* effect size correlation coefficients

^a,b^No significant difference between day-segments with equal superscript letters. All other combinations show a significant difference, *P* < 0.05

^c^Values were converted to 3 h averages

Both the quantitative (Table 3) and graphical analyses (Fig. 1b) of the behavioral domains of PA and SB reveal that during the various day-segments active and inactive behaviors go into competition with each other. Besides sleep (42 %), the dominant behavioral domains during the morning (6:00–9:00) were CADL (21 %), school (14 %) and motor travel (6 %). Of all active domains only active travel (5 %) had a meaningful contribution. The dominant behavioral domain during the early evening (15:00–18:00) was school, as most schools in Flanders end between 15:00 and 16:00. For early-evening leisure time, active travel (8 %) was the most contributing active domain. The inactive-leisure domains that were most apparent were screentime (12 %), homework (10 %), motor travel (8 %) and other inactive leisure activities (8 %). During the late evening (18:00–21:00) inactive behaviors were more dominant compared to active behaviors, 54 % of total time-use was spent at sedentary-intensity activity. The prominent inactive behavior during the late evening was screentime (31 %). Sport participation was the dominant active behavior during the late evening (8 %).

Table 4 shows the PA parameters and comparison between day-segments on Wednesdays. Again, the time variables from Table 4 represent the area under the curve in Fig. 2a (PA intensities) and 2b (behavioral domains) expressed in minutes per day-segment. Because the period before noon on Wednesdays does not differ from regular weekdays, only the results for the afternoon (12:00–15:00), early evening (15:00–18:00) and late evening (18:00–21:00) section are presented in Table 4.

**Table 4:** Comparison of physical activity and sedentary behavior between day-segments on Wednesdays

	Afternoon		Early evening		Late evening		Model	ES-*r*
	(12:00–15:00)		(15:00–18:00)		(18:00–21:00)		*P*-value	
	Mean (SD)	%	Mean (SD)	%	Mean (SD)	%		
PAL (MET_SWM_)	2.47 (0.71)^a^		2.69 (1.0)^b^		2.57 (1.17)^a,b^		0.04	0.12
Steps (n)	2439 (1504)^a^		2892 (2217)		2430 (2480)^a^		0.02	0.14
Time spent at different intensity levels								
Sedentary (min)	81 (36)^a,b^	45	75 (41)^a^	42	87 (44)^b^	48	0.01	0.16
Light (min)	84 (31)^a^	47	86 (34)^a^	48	70 (34)	39	<0.001	0.30
Moderate (min)	9 (9)^a^	5	11 (14)^a^	6	10 (14)^a^	5	0.10	0.10
Vigorous (min)	4 (7)	2	6 (11)^a^	3	8 (16)^a^	4	0.002	0.17
Sleep (min)	2 (15)^a^	1	2 (14)^a^	1	6 (18)	4	<0.001	0.22
Time spent at behavioral domains								
School (min)	7 (14)	4	0 (0)^a^	0	0 (0)^a^	0	<0.001	0.43
Sport (min)	7 (21)	4	26 (42)^a^	15	26 (41)^a^	15	<0.001	0.29
Active leisure (min)	11 (24)^a^	6	20 (33)	11	7 (19)^a^	4	<0.001	0.31
Active travel (min)	12 (18)	7	5 (12)	3	3 (7)	1	<0.001	0.36
Homework (min)	19 (26)^a^	11	18 (31)^a^	10	8 (19)	5	<0.001	0.24
Screentime (min)	24 (38)^a^	13	24 (36)^a^	14	48 (48)	27	<0.001	0.34
Inactive leisure (min)	22 (33)^a^	12	29 (41)^a^	16	23 (38)^a^	13	0.09	0.11
Motor travel (min)	15 (21)^a^	8	11 (21)^a^	6	6 (13)	4	<0.001	0.25
CADL (min)	31 (24)	17	16 (22)	9	38 (28)	21	<0.001	0.42
Other (min)	31 (32)^a^	17	30 (31)^a^	17	15 (21)	8	<0.001	0.40

Results are presented as means and standard deviations (SD).

^a,b^No significant difference between day-segments with equal superscript letters. All other combinations show a significant difference, *P* < 0.05

Based on PAL and number of steps, the afternoon was less active compared with the early evening section. With respect to the behavioral domains, all the inactive domains were more dominant during the afternoon day-segment compared with the active domains; active travel (7 %) was the main active behavior. During the early evening, the inactive domains that contributed most were inactive leisure (16 %) and screentime (14 %). Sports (15 %) and active leisure (11 %) were the most contributing active domains. Screentime (27 %), CADL (21 %) and sports (15 %) were the dominant domains during the late evening segment.

## Discussion

This study is the first to describe and investigate the temporal pattern of intensity- and domain-specific PA and SB in 10–14 year-old children. In the present study, sex and school-stage differences in activity levels were determined and the temporal pattern was examined during different segments of a typical weekday. The analysis of the temporal pattern revealed when specific activity behaviors typically take place during the course of the day and identified when active and sedentary behaviors compete with each other. Consequently, health professionals might employ these new insights to develop more effective PA interventions.

With regard the comparison of weekday levels of PA and SB, boys showed a higher PAL, more steps, more moderate PA, more vigorous PA and less sedentary behavior. These results confirm the well known gender difference in PA and SB, as epidemiological studies have consistently reported lower activity levels in girls compared with boys across all ages [14, 15, 33, 39]. In contrast, the expected higher activity level in primary school compared with secondary school could not be confirmed. No significant difference was found for PAL and secondary school children even outperformed primary school children with regard to moderate and vigorous PA. Nevertheless, primary school children showed higher levels of light-intensity PA and lower levels of sedentary time. The higher level of MVPA in secondary school children is somewhat surprising as several longitudinal studies have observed decreasing activity levels after the school transition [5, 40]. However, two other studies also found an increase in MVPA after the transition to secondary school [10, 41]. A more detailed comparison of activity levels between sexes and school stages for the current sample can be found in a previous study by De Baere et al. [22].

Despite these differences in activity levels, the graphs of the temporal pattern of PA and SB were very similar for both sexes and school stages. No differences with regard to the succession of activity peaks were observed, only the magnitude of the activity peaks showed some dissimilarity between the sexes and school stages. Riddoch et al. [33] also observed a very similar temporal pattern of accelerometer activity counts for boys and girls in their study on 11 year-old English school children. The explanation for the similar activity patterns of the subgroups is probably the fact that their school-schedules are practically identical. Moreover, day schedules in the family setting are unlikely to change when children make the transition from primary to secondary school. As a consequence, further analysis of the temporal pattern was executed for the total sample only. The analysis of the segmented temporal patterns revealed that during the morning segment (6:00–9:00), active travel was the only active domain that contributed substantially (5 %) to the total time-use of our sample. School-related active travel has already shown potential to contribute substantially to the overall activity levels during school days [42]. Since all children need to commute to school at more or less the same time, active travel competed directly against motorized travel. Due to long commuting distances, not all children have the opportunity to travel to school on foot or by bike [43]. For children that live within a reasonable distance of school, however, there is still room for improvement with respect to other barriers that limit active travel to school. The main barriers for this group are the parental safety concerns, including concerns with regard to both the physical (e.g., traffic safety, availability of sidewalks and bike lanes) and social environment (e.g., neighborhood safety) of the travel route [43]. By improving these environmental factors the proportion of children that choose active above motorized travel might increase [44].

The school segment (9:00–15:00) was, together with the early evening (15:00–18:00), the most active day-segment in our sample (PAL of 2.49 MET_SWM_ and 2.51 MET_SWM_ respectively). This result is somewhat surprising, as school days are characterized by substantial periods of institutionalized sitting during the theoretic classes. Our results mostly correspond with a study by Telford at al. [39], wherein more sedentary time was observed outside than during school time and almost no difference was found for in and out of school MVPA. Because of the substantial proportion of time spent in theoretic classes, activity levels during the school hours are limitedly amenable to improvement [45]. The moments that are improvable during school are the recess breaks and the lunch breaks. These free moments provide important opportunities for children to engage in moderate-to-vigorous PA (MVPA) through active play, as several PA-stimulating environmental factors are available (e.g., being outdoors, presence of peers and friends, relatively safe location) [46, 47]. An average of 7 % of the total time-use of our sample during school was spent at moderate and vigorous intensity. A large European study on PA and SB during school hours in a sample of 1025 children from various countries reported that only 5 % of the total school time was spent at MVPA [48]. School administrations should aim to increase the percentage of MVPA during recess, for example by providing access to school facilities during recess, provide portable play-ground equipment, create playground marking, introduce rotation of playground use and teach playground supervisors to promote PA rather than suppress it [46, 49–51]. The graphical analysis of the temporal pattern (Fig. 1a) showed different peaks and dips with intervals of approximately one hour for sedentary, light and moderate intensity activities during the school segment (9:00–15:00). The peaks for light and moderate intensity activity represent the PA during lunch breaks (i.e., largest activity peak), recess breaks (i.e., medium activity peaks) and the walking transitions between classrooms (i.e., smaller activity peaks). These hourly walking transitions are typically observed in secondary school children (see Additional files 3 and 4), since different classes often take place in specialized subject classrooms. In contrast, most of the classes in primary school take place in one and the same classroom. Clearly, the active transitions between different subject classrooms in secondary school help to interrupt periods of class-related sedentary behavior. Breaking longer periods of sedentary behavior by light-intensity activity during the school hours is known to exert positive effects with regard to musculoskeletal and metabolic health [52]. Therefore, primary schools might improve the activity behavior of their pupils by introducing short active breaks between theoretic classes.

During the early evening segment (15:00–18:00), once more the domains of active (8 %) and motorized travel (8 %) competed directly with each other. Furthermore, the inactive domains of screentime (12 %), homework (10 %) and inactive-leisure (8 %) were all more prominent compared to the active domains of sport (4 %) and active leisure (3 %). In literature, the after-school period is often referred to as the ‘critical hours’ for PA and SB, because children are freed from the constraints of the school environment and there is still enough daylight to engage in outdoor play or sport activities [45, 53]. Moreover, O’Connor et al. [54] showed that the activity level during the two hours immediately after school were predictive for the overall activity levels of children. Our results, however, showed that Flemish children were unable to take advantage of the PA opportunities during the early evening, as the competition between behaviors resulted in a clear dominance of sedentary activities. The graphical analysis of the temporal pattern showed the highest peak for homework during the early evening segment. Previous research has identified homework as a significant barrier for engagement in leisure-time PA [55, 56]. A lot of parents typically tell their offspring to do their homework immediately after school hours. Consequently, homework competes directly against sports activities and outdoor play during the most optimal timeframe for leisure-time PA. Few would advocate the replacement of homework by PA behavior, but the positioning of homework within the day schedule of children could be optimized to create a more PA-stimulating after-school period. In addition, research has shown that PA is associated with improved performance on cognitive tasks that demand concentration, attention and memory [57]. As a result, academic performance might even improve when children are encouraged to do their homework following a session of PA. Recently, also the concept of active homework has come to the attention, including strategies to reduce sitting time while completing homework tasks [58]. Introducing extra-curricular PA at school within a school-community partnership has proven to ameliorate children’s PA levels and health-related aspects [59–62] and might also be a solution for the homework-before-leisure mentality. Moreover, research on a sample of Flemish primary school children indicated that when extra-curricular PA was offered at school, the majority of children participated. Even two-thirds of the children that were not engaged in community sports were reached through the extra-curricular PA program [63].

The segment of the late evening (18:00–21:00) in our sample was dominated by sedentary activities, with screentime as the major contributor. Around 20:30, screen-based activities peaked at 48 % of all participants’ behaviors. Similar to the results from the study on the temporal pattern of PA and SB in children by Biddle et al. [20], screentime showed to be prominent throughout the whole evening, starting immediately after school until the end of the day. Introducing extra-curricular PA at school could help reduce the amount of screentime during the early evening. As a consequence, homework would compete directly against screentime later on the evening instead of competing against active behaviors during the ‘critical’ after-school period. After 17:00, PA behavior becomes less likely due to limited daylight, which restricts outdoor play and active travel [45].

The temporal pattern on Wednesday was different from other weekdays because of a shorter school segment (9:00–12:00). The activity pattern during the afternoon segment (12:00–15:00) on Wednesdays was very similar to the early evening segment (15:00–18:00) on other weekdays. The majority of sport (15 % of total time-use) and active-leisure activities (11 %) were situated during the early-evening segment, continuing during the first half of the late-evening segment (15 % and 4 % respectively). Since school is finished around noon on Wednesday, Flemish children have more space for leisure activities. Although the inactive behaviors (i.e., combination of screentime, homework and inactive leisure-time) were still more prominent, children were more engaged in sports and other active-leisure activities on Wednesday compared to other weekdays. Apparently, the longer leisure period on Wednesdays provides additional PA opportunities. For that reason, sport clubs in Flanders typically schedule their activities on Wednesdays.

The insight in the temporal patterns of activity behavior in children presented in this study, provide opportunities for a new approach on PA promotion. By reorganizing parts of the typical day schedule, children might be involuntarily guided towards more active and less sedentary lifestyles. Especially a reorganization of the period immediately after school has the potential to enhance activity levels of children on weekdays, for example by encouraging partnerships between schools and local sport clubs to provide qualitative extracurricular sports activities at school. Moreover, health professionals should focus on the moments when sedentary and active behaviors compete directly against each other (e.g., school transport) and create an environment that makes it more convenient for children and their parents to choose for the active alternative. Our study focused exclusively on weekday temporal patterns, because a more or less structured time-frame is needed to obtain valuable information from the temporal patterns on a group level. The school-day schedules of children offer such an organized framework, whereas patterns on weekend-days are very divers and less organized [33]. The fact that weekdays have a structured temporal pattern makes them also susceptible for improvement on a group level through PA interventions.

The major strength of this study is the assessment method that was employed to answer the research questions. By combining accelerometry through the SenseWear Mini Armband with registration of activity type through an electronic activity diary, we simultaneously captured all dimensions of PA behavior in children on a 24 h basis. As a consequence, we were able to investigate both the temporal pattern of activity intensities as well as the temporal pattern of the behavioral domains of PA and SB. Moreover, the electronic activity diary ruled out bias associated with self-report recall, as detailed activity information was registered in real-time into the device [28]. Finally, distinction between various sedentary behaviors was made. Beside the commonly investigated domain of screentime (i.e., watching television and playing computer games) we also included homework, motor travel, CADL and other inactive-leisure activities.

Although valuable insights in the temporal pattern of activity behaviors are provided, this study is not without limitations. First, the variability in PA and SB should be taken into account when interpreting the study outcomes. Our results demonstrated that PA levels can differ substantially between individuals. As a consequence, the interventions suggested in this study are appropriate to ameliorate the average PA level of our target group, but might not be of the best interest of every individual. Therefore, health professionals should carefully identify a target population and focus on their specific needs and interests when developing PA interventions. Secondly, biological maturity is also an important aspect to consider given the age-range of our sample. The high degree of variability in activity behaviors can be attributed partly to the variability in maturity-related aspects of our sample. Furthermore, the comparison of PA levels between primary and secondary school children was based on cross-sectional data instead of longitudinal data. For future studies it would be interesting to investigate how temporal patterns of PA and SB evolve longitudinally throughout the young years. Finally, the self-report nature of the electronic activity diary should be considered, as the registration of activity type partly relies on the subjective judgment of the participants. The electronic activity diary is also a rather burdensome assessment method. Participants had to carry the device and register their activities for the entire 7-day period, possibly causing small involuntary changes in activity behavior by the children in our sample.

## Conclusions

This study provides a better insight in the temporal pattern of PA and SB in 10–14 year-old children on weekdays. Our study revealed that daily levels of PA and SB differed between sexes and school stages, however, the temporal succession of the activities demonstrated almost no dissimilarities between these groups. Furthermore, our data showed that different types of PA and SB go into competition with each other at several time-points during the day. For the development of effective PA promotion strategies, the temporal pattern can be crucial information, as it has a direct impact on PA and SB of children. By making adaptations to the typical day schedule of children (e.g., through the introduction of extra-curricular PA after school hours), their daily activity levels might improve.

## Additional files

Additional file 1:
**Temporal pattern of PA and SB in primary school boys.** Occurrences of the different intensity levels (Fig. 1a) and behavioral domains (Fig. 1b) as a function of time on regular weekdays in primary school boys. (PDF 394 kb) Additional file 2:
**Temporal pattern of PA and SB in primary school girls.** Occurrences of the different intensity levels (Fig. 1a) and behavioral domains (Fig. 1b) as a function of time on regular weekdays in primary school girls. (PDF 393 kb) Additional file 3:
**Temporal pattern of PA and SB in secondary school boys.** Occurrences of the different intensity levels (Fig. 1a) and behavioral domains (Fig. 1b) as a function of time on regular weekdays in secondary school boys. (PDF 410 kb) Additional file 4:
**Temporal pattern of PA and SB in secondary school girls.** Occurrences of the different intensity levels (Fig. 1a) and behavioral domains (Fig. 1b) as a function of time on regular weekdays in secondary school girls. (PDF 411 kb) Additional file 5:
**Supplementary figure on the temporal pattern of behavioral domains.** Occurrences of all separate behavioral domains of PA and SB as a function of time on regular weekdays (in color + larger format). CADL: common activities of daily life. (PDF 231 kb)

## Abbreviations

PA: Physical activity
SB: Sedentary behavior
PHV: Peak height velocity
SWM: SenseWear Mini Armband
PAL: Physical activity level
MET_SWM_: Metabolic equivalents of SenseWear Mini
CADL: Common activities of daily life
ANCOVA: Analysis of covariance
MVPA: Moderate-to-vigorous physical activity
