# Supplementary figures and images for: Temporal patterns of physical activity and sedentary behavior in 10–14 year-old children on weekdays
Source: BMC Public Health. 2015 Aug 19;15:791. doi: 10.1186/s12889-015-2093-7 (PMC4545696; doi:10.1186/s12889-015-2093-7)

**A**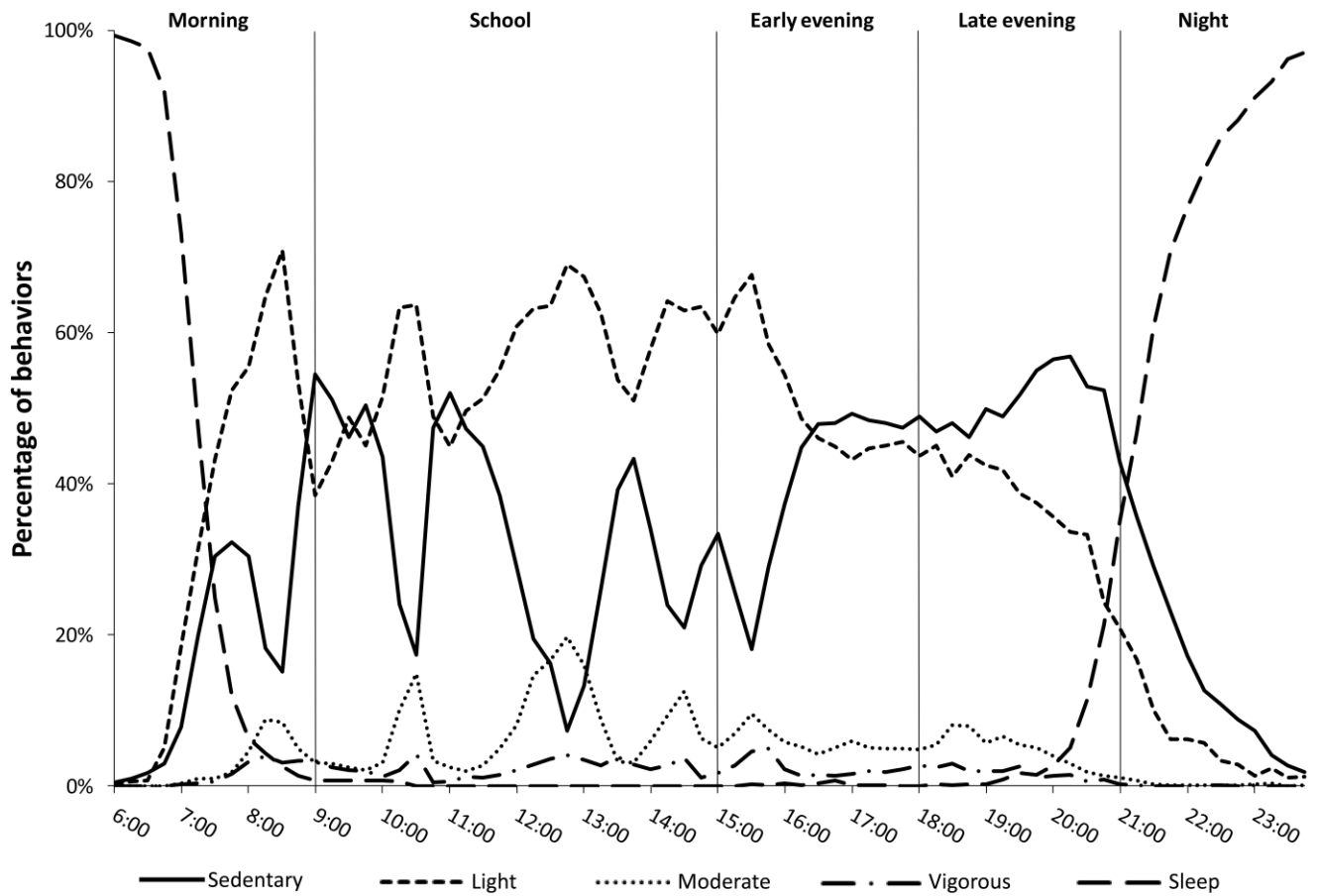**B**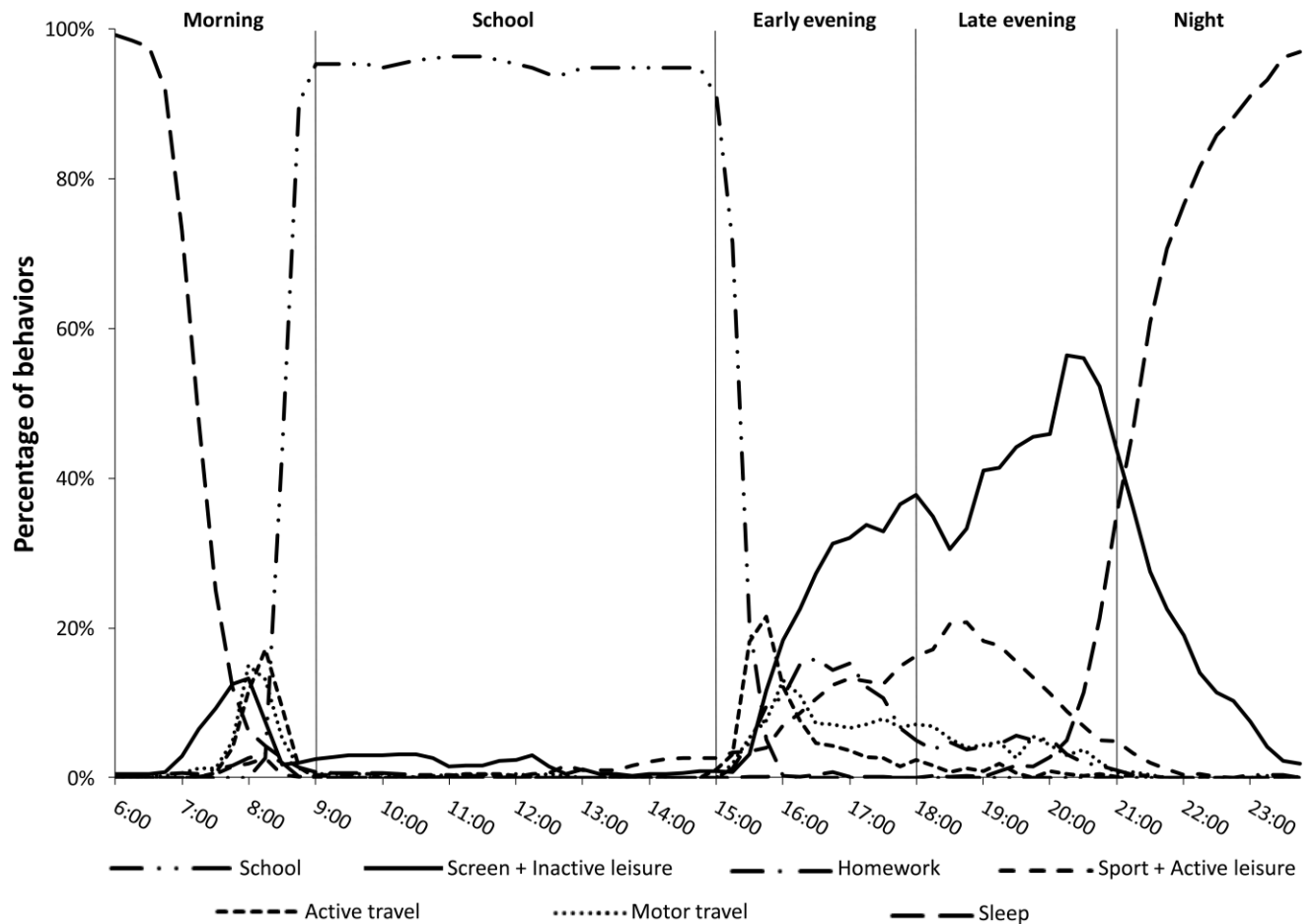

Supplement: Additional file 1: — Temporal pattern of PA and SB in primary school boys. Occurrences of the different intensity levels (Fig. 1a) and behavioral domains (Fig. 1b) as a function of time on regular weekdays in primary school boys. (PDF 394 kb) [file 12889_2015_2093_MOESM1_ESM.pdf]

**A**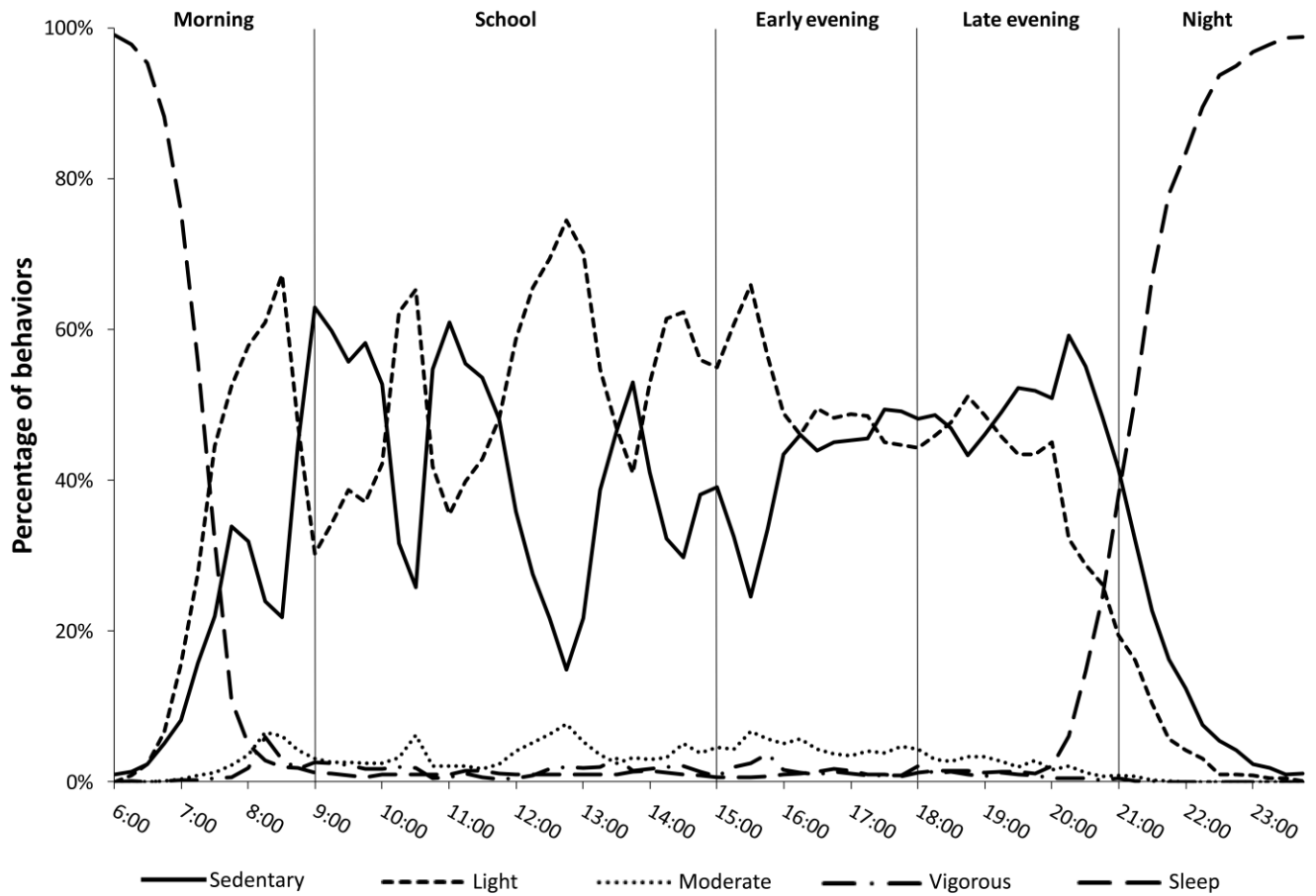**B**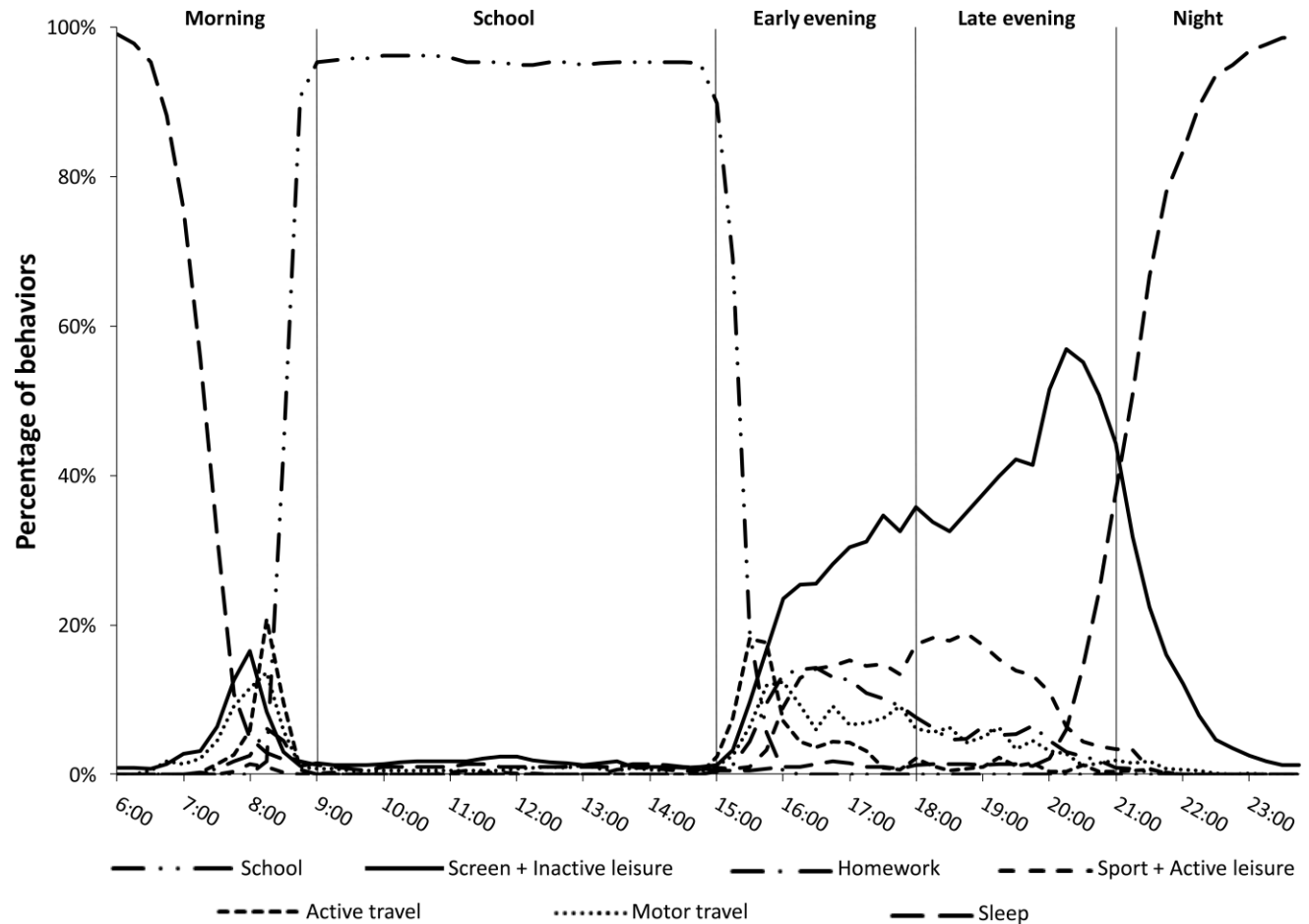

Supplement: Additional file 2: — Temporal pattern of PA and SB in primary school girls. Occurrences of the different intensity levels (Fig. 1a) and behavioral domains (Fig. 1b) as a function of time on regular weekdays in primary school girls. (PDF 393 kb) [file 12889_2015_2093_MOESM2_ESM.pdf]

**A**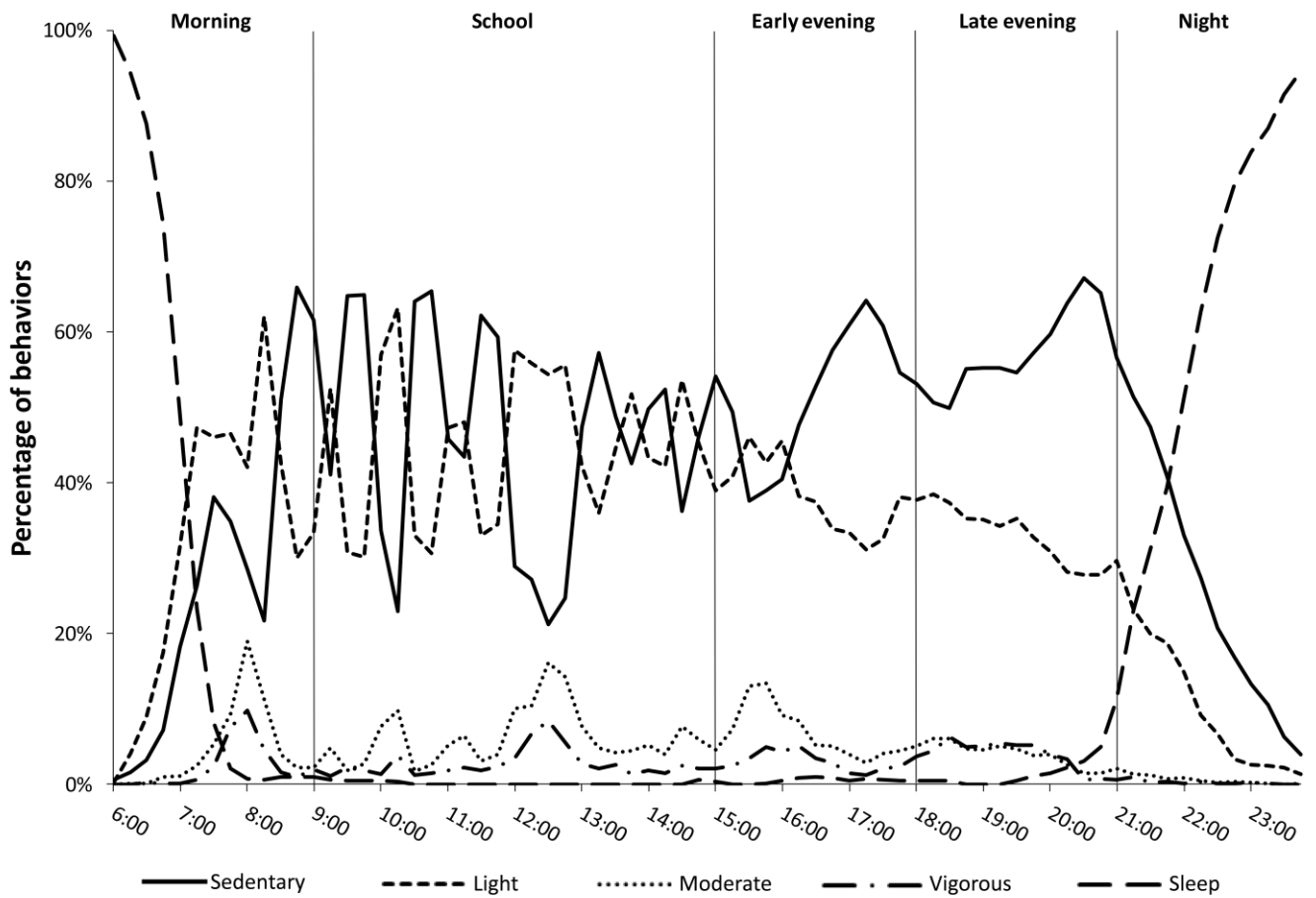**B**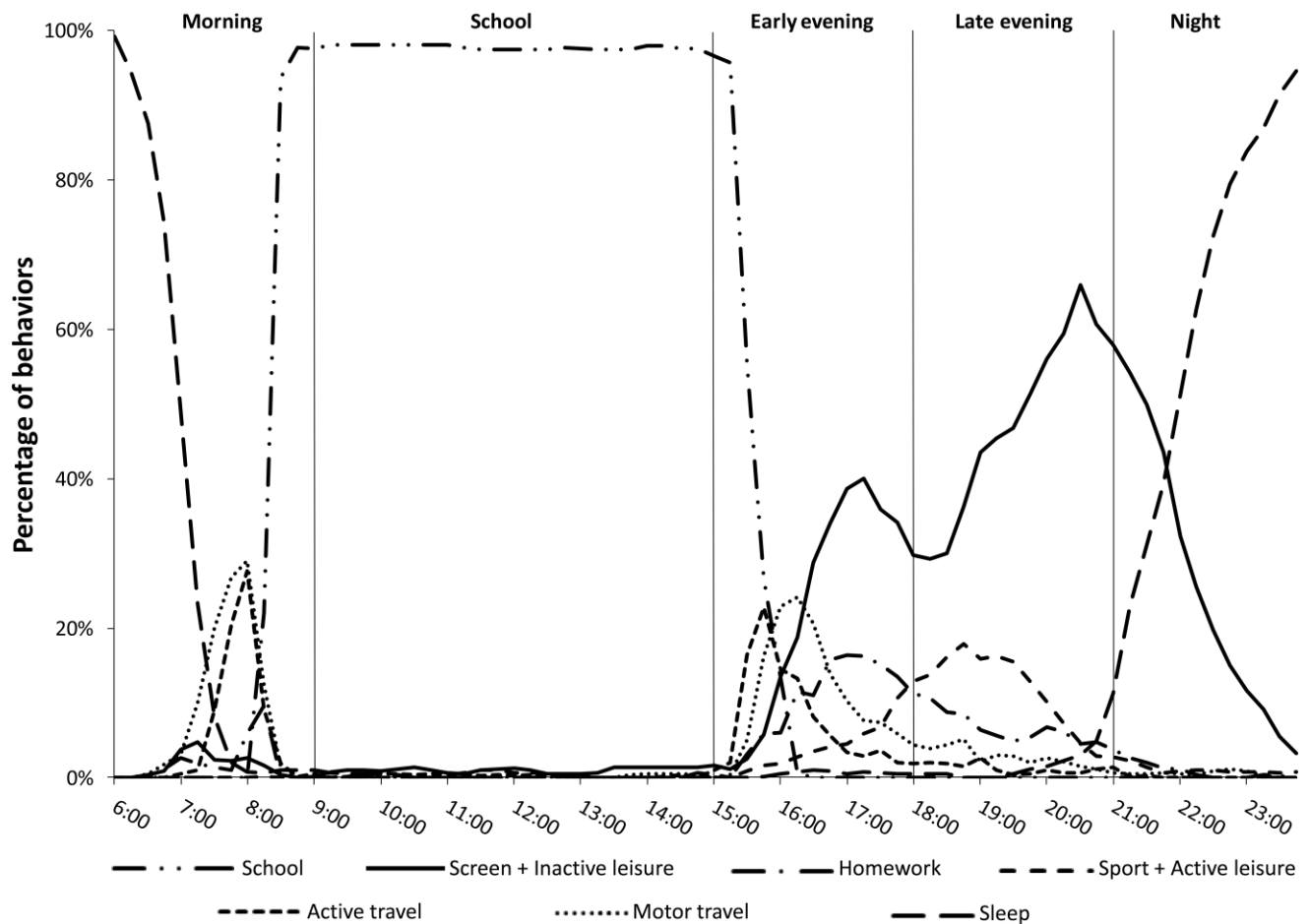

Supplement: Additional file 3: — Temporal pattern of PA and SB in secondary school boys. Occurrences of the different intensity levels (Fig. 1a) and behavioral domains (Fig. 1b) as a function of time on regular weekdays in secondary school boys. (PDF 410 kb) [file 12889_2015_2093_MOESM3_ESM.pdf]

**A**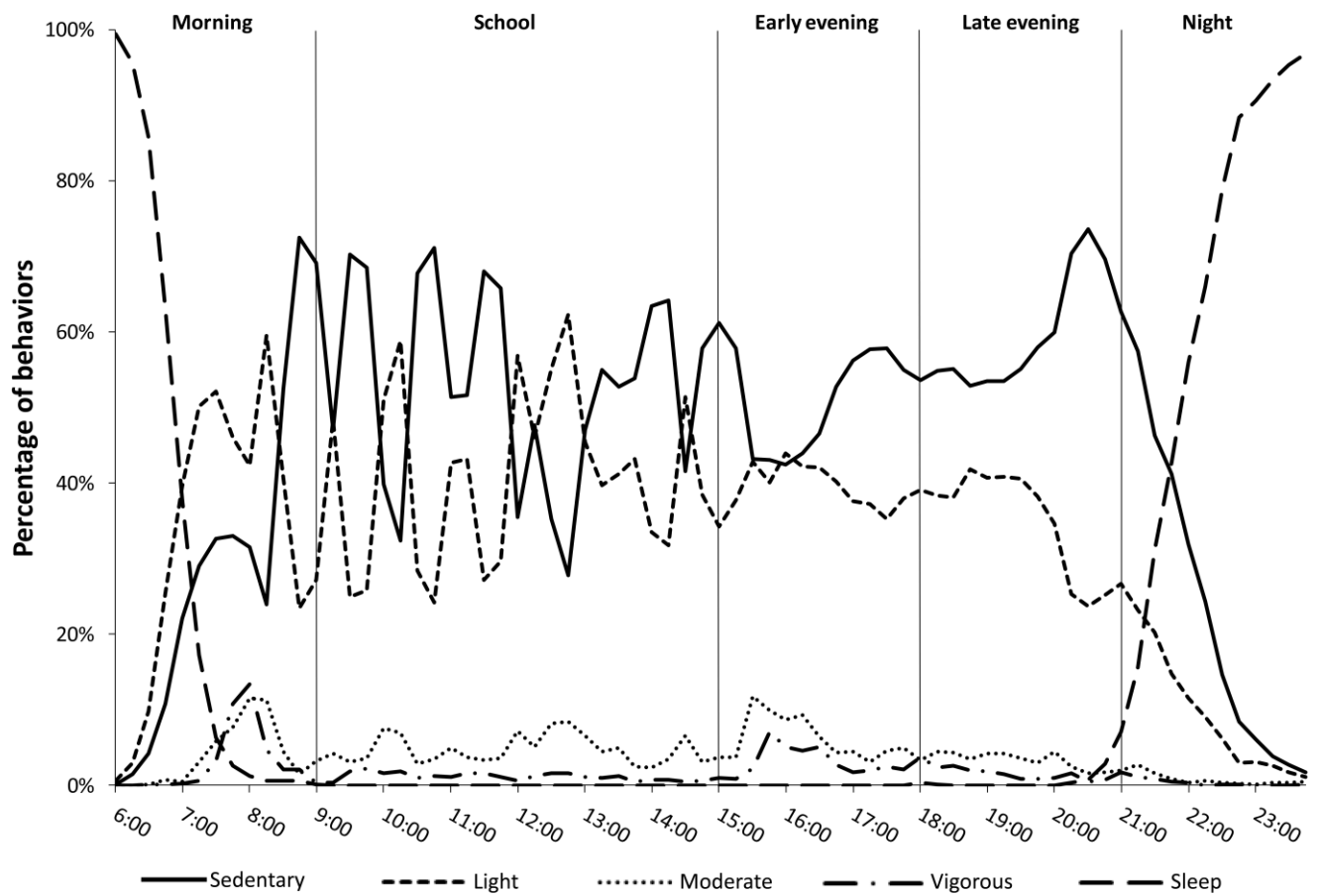**B**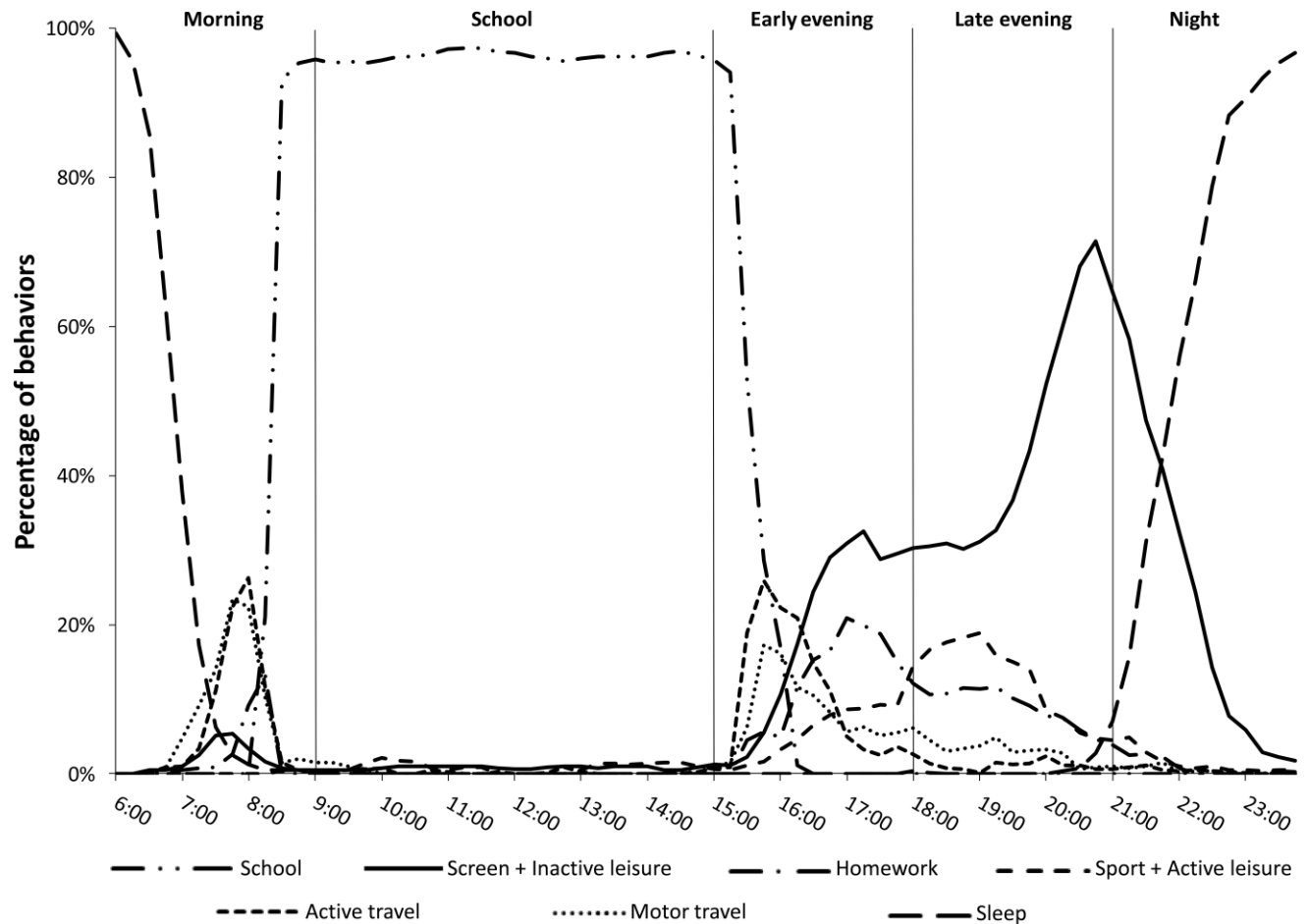

Supplement: Additional file 4: — Temporal pattern of PA and SB in secondary school girls. Occurrences of the different intensity levels (Fig. 1a) and behavioral domains (Fig. 1b) as a function of time on regular weekdays in secondary school girls. (PDF 411 kb) [file 12889_2015_2093_MOESM4_ESM.pdf]

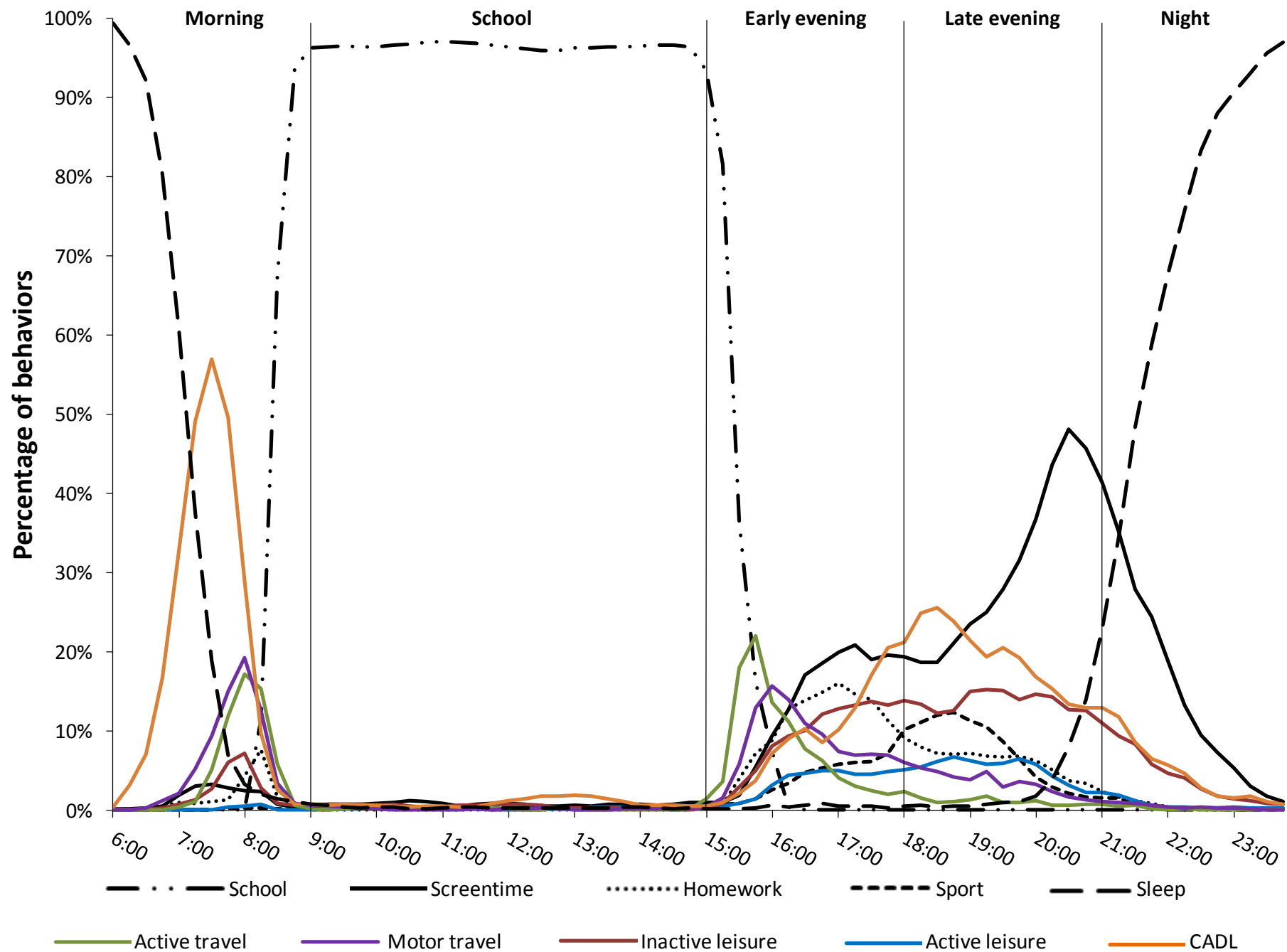

Supplement: Additional file 5: — Supplementary figure on the temporal pattern of behavioral domains. Occurrences of all separate behavioral domains of PA and SB as a function of time on regular weekdays (in color + larger format). CADL: common activities of daily life. (PDF 231 kb) [file 12889_2015_2093_MOESM5_ESM.pdf]
